# Laparoscopic repair of perforated peptic ulcer: a multicenter, propensity score matching analysis

**DOI:** 10.1186/s12893-022-01681-1

**Published:** 2022-06-16

**Authors:** Chang Woo Kim, Jong Wan Kim, Sang Nam Yoon, Bo Young Oh, Byung Mo Kang

**Affiliations:** 1 Department of Surgery, Ajou University School of Medicine, Suwon, Korea; 2 Department of Surgery, Dongtan Sacred Heart Hospital, Hallym University College of Medicine, Hwaseong, Korea; 3 Department of Surgery, Kangnam Sacred Heart Hospital, Hallym University College of Medicine, Seoul, Korea; 4 Department of Surgery, Hallym Sacred Heart Hospital, Hallym University College of Medicine, Anyang, Korea; 5 Department of Surgery, Chuncheon Sacred Heart Hospital, Hallym University College of Medicine, 77 Sakju-ro, 24253 Chuncheon, Korea

**Keywords:** Peptic ulcer, Peptic ulcer perforation, Laparoscopy, Complication, Propensity score

## Abstract

**Background:**

Perforated peptic ulcer (PPU) is a common emergency condition requiring surgery using laparoscopy or open repair of the perforated site. The aim of this study was to assess the role of laparoscopic surgery (LS) based on the safety and efficacy for PPU.

**Methods:**

Medical records of the consecutive patients who underwent LS or open surgery (OS) for PPU at five hospitals between January 2009 and December 2019 were retrospectively reviewed. After propensity score matching, short-term perioperative outcomes were compared between LS and OS in selected patients.

**Results:**

Among the 598 patients included in the analysis, OS was more frequently performed in patients with worse factors, including older age, a higher American Society of Anesthesiologists score, more alcohol use, longer symptom duration, a higher Boey score, a higher serum C-reactive protein level, a lower serum albumin level, and a larger-diameter perforated site. After propensity score matching, 183 patients were included in each group; variables were well-balanced between-groups. Postoperative complications were not different between groups (24.6% LS group vs. 31.7% OS group, p = 0.131). However, postoperative length of hospital stay (10.03 vs. 12.53 days, respectively, p = 0.003) and postoperative time to liquid intake (3.75 vs. 5.26 days, p < 0.001) were shorter in the LS group.

**Conclusions:**

LS resulted in better functional recovery than OS and can be safely performed for treatment of PPU. When performed by experienced surgeons, LS is an alternative option, even for hemodynamically unstable patients.

## Background

The incidence of peptic ulcer disease has decreased with the use of appropriate treatment strategies against the most common etiologies, which are *Helicobacter pylori* and the use of nonsteroidal anti-inflammatory drugs [1]. Although there are other etiologies of peptic ulcer disease, eradication of *H. pylori* and use of proton pump inhibitors have contributed to curing the disease and prevention of complications such as bleeding and perforation.

Perforated peptic ulcer (PPU) is a common emergency condition requiring surgical intervention; mortality rates are as high as 30% [2]. Despite advances in medical treatments for peptic ulcer disease, PPU incidence has not significantly decreased [1, 3]. Spillage of gastric contents with bile acid into the intraperitoneal space causes localized or generalized peritonitis, which quickly leads to sepsis and a life-threatening condition. Early surgery using laparoscopic or open repair of the perforated site with proper sepsis management is essential for a good outcome [2, 4].

Open surgery (OS) has been used to treat PPU for the past decades. However, because laparoscopic surgery (LS) has advantages including a shorter incision length, less postoperative pain, and early recovery for various diseases [5–7], the first laparoscopic repair of PPU was reported by Mouret et al. [8]. Thereafter, retrospective studies found acceptable outcomes of LS use for PPU, but because of inconsistent results surgeons select a procedure based on personal preference [9–11]. A meta-analysis that included eight randomized controlled trials found that use of LS results in less early postoperative pain and lower wound infection rates [12–17]. However, each study had some limitations such as small numbers of patients, variable levels of surgical experience performing LS, and use of single-center data. The aim of this multicenter, large-scaled retrospective study was to assess the role of LS based on the safety and efficacy of laparoscopic primary repair for PPU. We compared short-term perioperative outcomes between LS and OS in patients with PPU.

## Methods

### Patients

**Fig. 1:**
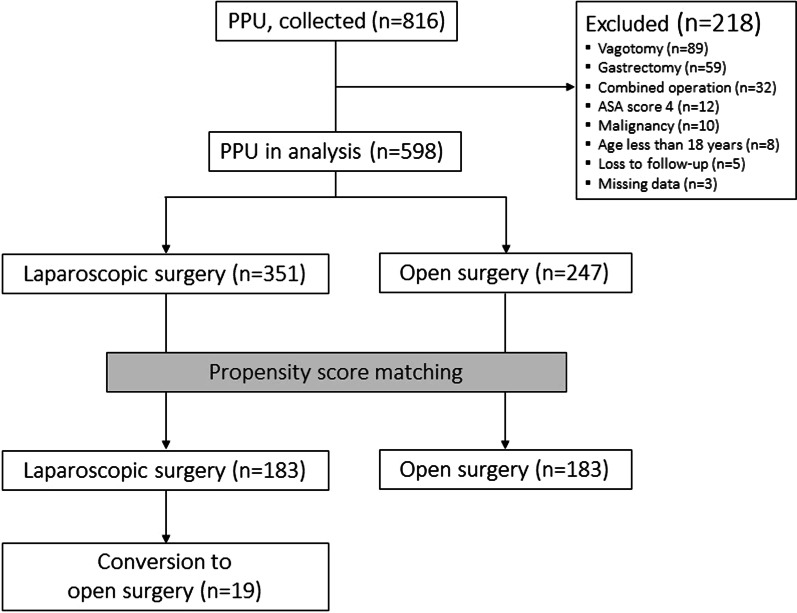
Study flow diagram. *PPU* perforated peptic ulcer, *ASA* American Society of Anesthesiologists

Medical records of the consecutive patients who underwent LS or OS for PPU at five university hospitals in South Korea between January 2009 to December 2019 were retrospectively reviewed. The study protocol was approved by the Institutional Review Board at each participating institution and written informed consent was waived due to the observational and retrospective nature of the study (Institutional Review Board of Kyung Hee University Hospital at Gangdong, Hallym University Dongtan Sacred Heart Hospital Institutional Review Board / Ethics Committee, Hallym University Kangnam Sacred Heart Hospital Institutional Review Board / Ethics Committee, Hallym University Sacred Heart Hospital Institutional Review Board / Ethics Committee and Hallym University Chucheon Sacred Heart Hospital Institutional Review Board / Ethics Committee). The study was conducted in accordance with the Declaration of Helsinki. Only patients who underwent primary closure for PPU were included during the study period. Exclusion criteria were patients with (1) any type of gastrectomy or gastrojejunostomy, (2) combined vagotomy, (3) combined surgery for other organs, (4) gastric malignancy, (5) age < 18 years, (6) an American Society of Anesthesiologists (ASA) score > 3, and (7) loss to follow-up after surgery. Among a total of 816 patients, data from 598 with PPU were included in the analysis after exclusion of 218 patients (Fig. 1).

## Surgical procedures

The diagnosis was confirmed based on a history of peptic ulcer or *H. pylori* infection or chronic use of ulcerogenic drugs, clinical symptoms (sudden onset of abdominal pain, fever, and chills) and signs (tenderness, rebound tenderness, and involuntary guarding found during an abdominal examination), and radiologic findings (e.g., intraperitoneal free air in upright abdominal radiographs, and intraperitoneal fluid collection and/or gastric or duodenal wall thickening revealed by abdominal computed tomography). Intravenous fluid resuscitation was performed and empirical antibiotics were administered. A urethral indwelling catheter was inserted to monitor for appropriate hydration. The operation method used (LS or OS) was chosen to achieve good outcomes within a short operation time based on careful consideration of factors including age of the patient, vital signs, PPU severity, history of previous abdominal surgery, and the surgeon’s preference. All participating surgeons had experience with > 200 cases of laparoscopic abdominal surgery before the study was performed.

After general endotracheal anesthesia, each patient was placed in a reverse Trendelenburg or lithotomy position. An upper midline incision was commonly used for OS. For LS, a 10 mm trocar was inserted for entry of the laparoscope in the periumbilical area; two or three 5-mm trocars were inserted in both lower quadrants for entry of the working instruments. The perforation site and degree of peritoneal contamination were revealed using intraperitoneal exploration. Primary repair of the perforated site was performed using interrupted or continuous suture techniques. Omentopexy or fibrin sealant application was performed according to the surgeon’s decision. A contaminated intraperitoneal cavity was irrigated using a warm saline solution. When the presence of an underlying malignancy was suspected at the perforated site, a biopsy was obtained from the site margin. Surgical drainage was inserted in the right subhepatic area, if needed. The wound was closed using layer-by-layer sutures. The diet was resumed after bowel movement confirmation, and the patient was discharged with tolerable pain that was controlled using oral medication. Details of the surgical procedures and perioperative management protocols used, such as proton pump inhibitor use, *H. pylori* eradication, postoperative enteral contrast imaging before diet resumption, diet schedule, criteria for discharge, and postoperative upper gastrointestinal endoscopy were based on the policies of the individual surgeons and institutions.

### Outcomes measured

Data were collected by reviewing medical records and were registered on a case reporting form used for the study. Variables for baseline characteristics included in the analysis were sex, age, body mass index (BMI), ASA score, history of previous abdominal surgery, current status of alcohol consumption and smoking, Boey score, complete blood count with differential, C-reactive protein (CRP), and serum albumin level at admission. Operative variables included operation time, operation method, PPU site and diameter, postoperative complications and mortality, time to functional recovery, and postoperative length of hospital stay (LOS). The Boey scoring system consisted of three risk factors for postoperative complications (concomitant severe medical illness, preoperative shock, and a duration of perforation > 24 h); each factor was given a score of 1 point, when positive. The Boey score for each patient was calculated based on the sum of points for each risk factor (score 0–3) and was used for PPU risk stratification [18]. Preoperative neutrophil-to-lymphocyte ratios (NLRs) and platelet-to-lymphocyte ratios (PLRs) were assessed as prognostic factors for peritonitis [19, 20]. The primary outcome was the 30-day postoperative complication rate. Severity of each postoperative complication was assessed using Clavien-Dindo classification criteria [21]. We clarified the definition of each postoperative complication in the study protocol. Wound infection or seroma was defined as presence of purulent or serous discharge on the surgical wound. Pneumonia was defined as symptoms of lower respiratory tract infection and radiologic evidence of pulmonary infiltration. Prolonged ileus was defined as the absence of gas passage and small bowel distention after postoperative day 5. Leakage was defined as operative or radiologic findings of one or more gross defects at the primary closure site. Intraoperative abscess was defined as abscess formation at any site in the abdomen unrelated to the leakage. Bleeding was defined as postoperative bleeding requiring blood transfusion. Voiding difficulty was defined as urinary retention requiring re-insertion of a urethral indwelling catheter.

### Statistical analyses

Continuous variables were compared using Student’s *t* tests. Categorical variables were compared using Pearson’s chi-square tests or Fisher’s exact tests. A P value was considered statistically significant when it was < 0.05. The propensity score was estimated based on sex, age, BMI, current alcohol consumption, smoking, and the Boey score using logistic regression modeling and matching at a 1:1 ratio. All statistical analyses were performed using Statistical Package for the Social Sciences software (SPSS, version 20.0, Chicago, IL, USA).

## Results

### Baseline characteristics

**Fig. 2:**
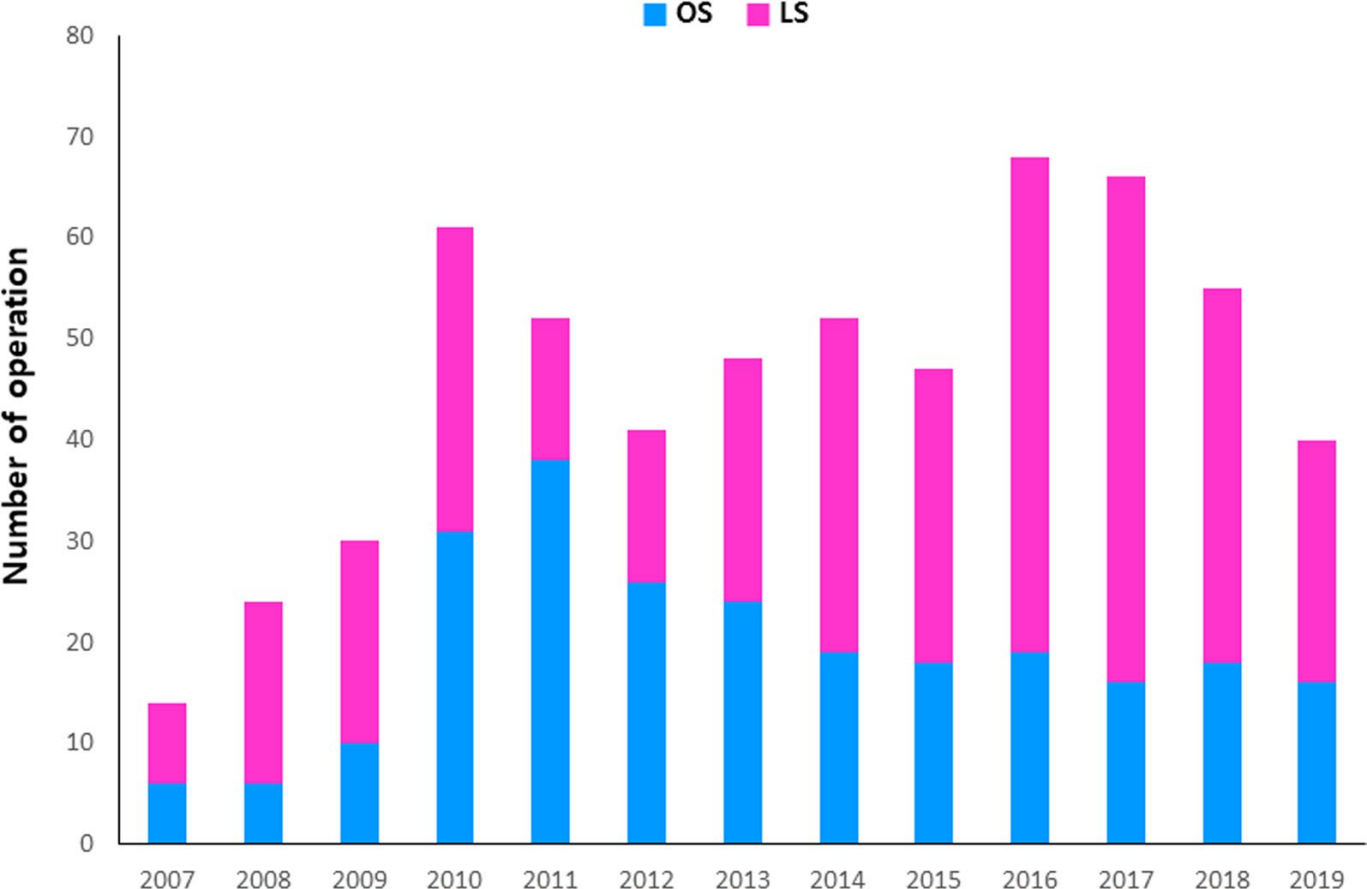
Number of operations. *OS* open surgery, *LS* laparoscopic surgery. Blue bar indicates the number of OSs and red bar indicates the number of LSs

Among the 598 patients with PPU included in the analysis, LS was performed in 351 patients and OS was performed in 247 patients (Table 1). The proportion of male patients was significantly lower (83.5% LS vs. 72.9% OS, p = 0.002), and the mean age was significantly older (52.64 years LS vs. 58.65 years OS, p < 0.001), in the OS group than in the LS group. The ASA score was higher in the OS group (p = 0.001). BMI and history of previous abdominal surgery were similar between the groups. A few variables that reflect disease severity were poorer in the OS group than in the LS group. Mean symptom duration between the onset of abdominal pain and surgery was significantly longer (14.57 h LS vs. 19.41 h OS, p = 0.021), and the mean Boey score was significantly higher (p < 0.001), in the OS group. Mean serum CRP was significantly higher (18.65 mg/L LS vs. 42.52 mg/L OS, p = 0.001) and mean serum albumin level was significantly lower (4.00 g/dL LS vs. 3.83 g/dL OS, p = 0.001) in the OS group. White blood cell count, neutrophil count, hemoglobin level, and NLR and PLR were not different between-groups. Mean diameter of the perforated site was significantly larger in the OS group (7.77 mm LS vs. 8.88 mm OS, p = 0.048). Figure 2 presents results for OSs and LSs and proportions for LSs per year. Since 2010, when the total number of operations was > 40 cases, the proportion of LSs for PPU tended to gradually increase.

**Table 1:** Baseline characteristics

	Total Cohort			Matched Cohort		
	LS (n = 351)	OS (n = 247)	P value	LS (n = 183)	OS (n = 183)	P value
Male sex	293 (83.5%)	180 (72.9%)	0.002	138 (75.4%)	140 (76.5%)	0.807
Age, year	52.64 (18–97)	58.65 (19–92)	< 0.001	56.03 (18–97)	55.94 (19–92)	0.960
BMI, kg/m^2^	21.83 (14.30–33.30)	21.84 (12.27–33.30)	0.992	21.67 (14.30–33.30)	21.82 (14.47–33.30)	0.654
ASA score, n (%)			0.001			0.951
1	218 (62.1%)	122 (49.4%)		100 (54.6%)	100 (54.6%)	
2	90 (25.6%)	67 (27.1%)		50 (27.3%)	48 (26.2%)	
3	43 (12.3%)	58 (23.5%)		33 (18.0%)	35 (19.1%)	
Previous abdominal surgery	37 (10.5%)	32 (13.0%)	0.363	26 (14.2%)	27 (14.8%)	0.882
Current alcohol consumption	208 (59.3%)	103 (41.9%)	< 0.001	86 (47.0%)	89 (48.6%)	0.754
Current smoker	185 (52.7%)	114 (46.3%)	0.126	94 (51.4%)	92 (50.3%)	0.834
Symptom duration before operation, hour	14.57 (2–126)	19.41 (3–224)	0.021	16.24 (2–126)	14.60 (3–80)	0.395
Boey score			< 0.001			0.892
0	273 (78.0%)	147 (59.5%)		128 (69.9%)	125 (68.3%)	
1	64 (18.3%)	70 (28.3%)		45 (24.6%)	46 (25.1%)	
2	13 (3.7%)	27 (10.9%)		10 (5.5%)	12 (6.6%)	
3	0 (0%)	3 (1.2%)		0 (0%)	0 (0%)	
White blood cell count, 10^9^/L	11.66 (1.90–47.70)	12.00 (1.29–64.70)	0.520	11.95 (1.90–47.70)	11.55 (1.50–31.10)	0.499
Neutrophil count, 10^9^/L	9.50 (1.04–42.93)	9.91 (1.00–61.98)	0.424	9.81 (1.04–42.93)	9.36 (1.17–28.77)	0.415
Hemoglobin, g/dL	13.82 (4.1–19.7)	13.69 (5.0–20.0)	0.566	13.33 (4.1–19.7)	13.74 (5.0–20.0)	0.145
CRP, mg/L	18.65 (0.01–433.80)	42.52 (0.06–403.20)	0.001	24.35 (0.01–433.80)	32.21 (0.06–368.95)	0.317
NLR	10.84 (0.38–92.00)	11.38 (0.87–67.35)	0.565	10.91 (0.97–61.00)	10.06 (0.87–67.35)	0.421
PLR	307.01 (0.83–3757.4)	348.54 (44.97–1835)	0.141	301.18 (0.83–3757.4)	316.83 (44.97–1835)	0.641
Serum albumin, g/dL	4.00 (1.7–5.1)	3.83 (1.5–4.9)	0.001	3.95 (2.3–5.0)	3.93 (1.9–4.9)	0.807
Site of PPU			0.537			> 0.999
Stomach	147 (41.9%)	108 (43.7%)		78 (42.6%)	78 (42.6%)	
Duodenum	203 (57.8%)	139 (56.3%)		105 (57.4%)	105 (57.4%)	
Unknown	1 (0.3%)	0 (0%)		0 (0%)	0 (0%)	
Diameter of perforation, mm	7.77 (1–30)	8.88 (1–50)	0.048	8.18 (1–30)	8.71 (1–50)	0.457

Results are presented as number (%) of patients or mean (range)

*LS* laparoscopic surgery, *OS* open surgery, *BMI* body mass index, *ASA* American Society of Anesthesiologists, *CRP* C-reactive protein, *NLR* neutrophil-to-lymphocyte ratio, *PLR* platelet-to-lymphocyte ratio, *PPU* perforated peptic ulcer

After propensity score matching, 183 patients were included in the LS group and in the OS group. The matched cohort analysis revealed that after matching, the variables found to be significantly different before matching (i.e., sex, age, ASA score, alcohol and smoking habits, symptom duration, Boey score, serum CRP, albumin level, diameter of perforated site) were well-balanced between both groups.

### Operative outcomes

The results for operative outcomes after matching are presented in Table 2. Mean operation time was not significantly different between-groups (78.17 min LS vs. 82.84 min OS, p = 0.217). Omentopexy was performed equally in both groups, but fibrin sealant was used more frequently in the OS group (11.5% LS vs. 26.8% OS, p < 0.001). Conversion to OS was performed in 19 (10.4%) patients in the LS group. The reasons for open conversion were difficulty locating the perforated site (6 patients), difficulty making the operative field (5 patients), inflammatory adhesion (3 patients), a large defect (3 patients), friable tissue (1 patient), and unknown cause (1 patient). Surgical drainage was more frequently inserted in the LS group (100% LS vs. 93.4% OS, p < 0.001). The postoperative LOS (10.03 days LS vs. 12.53 days OS, p = 0.003), time to liquid intake (3.75 days LS vs. 5.26 days OS, p < 0.001), and time to soft diet intake (5.47 days LS vs. 7.02 days OS, p < 0.001) were significantly shorter in the LS group.

**Table 2:** Operative outcomes

	LS (n = 183)	OS (n = 183)	P value
Operation time, minute	78.17 (28–390)	82.84 (30–210)	0.217
Omentopexy	152 (83.1%)	152 (83.1%)	> 0.999
Fibrin sealant	21 (11.5%)	49 (26.8%)	< 0.001
Conversion to open surgery	19 (10.4%)		
	Difficulty in localization (6)		
	Difficulty in making operative field (5)		
	Inflammatory adhesion (3)		
	Large defect (3)		
	Friable tissue (1)		
	Unknown (1)		
Drainage insertion	183 (100%)	171 (93.4%)	< 0.001
Postoperative LOS, day	10.03 (3–43)	12.53 (1–89)	0.003
Time to start liquid intake, day	3.75 (1–12)	5.26 (1–22)	< 0.001
Time to resuming soft diet intake, day	5.47 (2–30)	7.02 (3–23)	< 0.001
Time to removal of drainage, day	6.61 (2–23)	7.45 (4–20)	0.076
30–day postoperative complication	45 (24.6%)	58 (31.7%)	0.131
Mortality	7 (3.8%)	9 (4.9%)	0.609

Results are presented as number (%) of patients or mean (range)

*LS* laparoscopic surgery, *OS* open surgery, *LOS* length of stay

The overall rate of 30-day postoperative complications, the primary study outcome, was slightly lower in the LS group than in the OS group, but this difference was not statistically significant (24.6% LS vs. 31.7% OS, p = 0.131). The most common complications were wound-related (3.83% LS vs. 8.20% OS, Table 3). Severity of postoperative complications, stratified using the Clavien-Dindo classification, was not significantly different between-groups. The mortality rate was also similar between-groups (3.8% LS vs. 4.9% OS, p = 0.609) (Table 2).

**Table 3:** 30-day postoperative complications according to the Clavien-Dindo classification

	LS (n = 183)	OS (n = 183)	P value
Grade			0.352
Grade I	2 (1.1%)	7 (3.8%)	
Wound infection	1	5	
Wound seroma	1	2	
Grade II	29 (15.8%)	29 (15.8%)	
Wound infection	2	4	
Pneumonia	6	10	
Prolonged ileus	5	2	
Intraperitoneal abscess	4	3	
Bleeding	0	4	
FUO	2	0	
Voiding difficulty	0	3	
Pleural effusion	2	0	
Arrhythmia	2	0	
CVA	2	0	
Delirium	0	2	
Others	4	1	
Grade IIIa	6 (3.3%)	11 (6.0%)	
Wound infection	2	1	
Pleural effusion	2	4	
Intraperitoneal abscess	1	3	
AKI	1	2	
Others	0	1	
Grade IIIb	3 (1.6%)	4 (2.2%)	
Wound dehiscence	1	3	
Leakage	1	1	
Intraperitoneal abscess	1	0	
Grade IV	5 (2.7%)	7 (3.8%)	
Sepsis	3	4	
Pneumonia	2	2	
Leakage	0	1	

Results are presented as number of patients

*LS* laparoscopic surgery, *OS* open surgery, *FUO* fever of unknown origin, *CVA* cerebrovascular accident, *AKI* acute kidney injury

## Discussion

As laparoscopic techniques have been applied to various diseases and more superior perioperative outcomes than with OS have been found, many studies have reported safety and efficacy findings of LS for the treatment of PPU [9, 15, 16]. Concerns about LS such as a longer operation time, insufficient lavage, and possible repair site leakage seem to be on the decline as LS experience and data have accumulated. A meta-analysis found that LS for PPU has similar short-term outcomes, but less early postoperative pain and wound infection [3, 12]. We also found no difference in postoperative complication rates, the primary endpoint of this case-matched study, between the LS and OS groups. Consistent with previous studies, we also found that LS for PPU yielded better functional recovery than OS, including more rapid diet resumption and a shorter postoperative LOS [10, 16].

The preoperative condition of a patient can affect the decision about the type of surgery used. Traditionally, a patient with poor condition and severe disease would undergo OS rather than LS. Patients with severe peritonitis with a large amount of purulent fluid or with fecal material in the intraperitoneal cavity usually require rapid and massive irrigation rather than use of laparoscopic irrigation, which involves a small-diameter opening and is a slow process. Carbon dioxide retention can occur via intraperitoneal CO_2_ insufflation during LS, which might affect an unstable patient’s outcome [22]. Therefore, during previous decades OS has been performed in patients with worse factors, including old age, higher ASA score, alcohol consumption, longer symptom duration, a higher Boey score, and poor laboratory results (e.g., for CRP and albumin). We also found significant differences in these variables before propensity score matching. Even though the surgeons in this study had experience using LS for abdominal disorders, patients with more severe conditions tended to undergo OS instead of LS for PPU before matching, especially during the early part of the study period. Therefore, all variables were balanced via matching to reduce the possibility of allocation bias.

Similar to previous meta-analyses that found similar or marginally shorter operation times for LS than OS, operation time was not different in this study (78.17 min LS vs. 82.84 min OS, P = 0.217) [3, 12]. The small difference (about 5 min) might be related to the combination of longer abdominal wall open and closure times, but a shorter primary repair time, of the perforated site in OS compared with LS. Omentopexy rates were same in both groups, but more fibrin sealant application was performed in the OS group than in the LS group. These variables can be affected by (1) the surgeon’s preference and (2) changes related to improvements in laparoscopic instruments and skills. Some investigators reported results using a fibrin glue application for the PPU site in the early 1990 s, but few reports have recently been published [8, 23]. Most fibrin sealant-applied cases were performed during the early period of this study.

Time to diet resumption was shorter in the LS group than the OS group; the subsequent LOS was also shorter. Earlier functional recovery from LS compared with OS has been found in previous studies [5, 7]. Patients who undergo LS for colorectal, gastric, and hepatobiliary disease experience less pain, faster resumption of normal bowel movements, and a shorter LOS than those who undergo OS. Previous studies have also reported similar results for PPU. Lau et al. found that the amount of analgesia required after LS is less than for OS, although the operation time is longer [13]. A randomized controlled trial performed by Bertleff and colleagues found that LS results in low pain scores and shorter hospital stays [16].

There were no between-group differences in specific Clavien-Dindo classification-based postoperative complication rates or in overall rates. The OS group tended to have more complications (24.6% LS vs. 31.7% OS, P = 0.131); this difference might be related to wound-associated complications (7 patients LS vs. 15 patients OS). Wound-associated complications occur more frequently after OS than after LS. Cirocchi et al. found that LS has a lower postoperative wound infection rate than OS for PPU [12].

Based on various outcomes that favor LS more than OS, we believe that LS is an alternative option for successful PPU treatment. Some authors think that LS should be performed only for hemodynamically stable patients [9]. However, we compared the patients’ Boey score data and found no differences associated with preoperative shock. Because of improvements in surgical skills and instruments for LS, better perioperative outcomes can be obtained by LS-experienced surgeons, even in patients who are hemodynamically unstable.

To our knowledge, this study is the largest to date to compare LS and OS for PPU. However, some limitations should be noted. First, the retrospective nature of the study might have affected the results. The nature of the disease, including patient instability, perforation size, and unclear origin of the perforation could have affected the results. Second, propensity score matching was performed to reduce allocation bias, but selection bias was still possible. Third, the analysis was not stratified by participating surgeon or institution; the operation method used could vary by the surgeon’s preference, technique, experience, and the institution’s policies and medical procedures offered. Moreover, most medical records did not reveal the reason LS or OS was chosen. Fourth, the participating surgeons were highly experienced laparoscopic surgeons who had each performed > 200 laparoscopic surgeries. Therefore, these results might be not reproducible in real medical practice where emergent surgery is required. Fifth, postoperative pain is one important variable used to examine efficacy of LS compared with OS. However, postoperative pain was not analyzed because medical records did not include the required data. Sixth, no long-term outcomes such as incisional hernia or recurrence of PPU were recorded because most patients visited the outpatient clinic only once or twice after surgery. Last, we believe the advantages of LS can benefit high-risk patients. However, because propensity matching excluded these patients, we could not examine this hypothesis about LS efficacy using this study design. In addition, patients with an ASA score = 4 were excluded from the analysis because postoperative outcomes of these patients could be predicted by the severity of underlying disease, rather than the surgical method used (OS vs. LS). This question should be examined using well-designed randomized controlled trials.

## Conclusions

There was no difference in terms of postoperative complications between the LS and OS groups. Rather, LS yielded better functional recovery than OS. LS can be safely performed for treatment of PPU. When performed by experienced surgeons, it can be an alternative option, even for hemodynamically unstable patients.

## Data Availability

The datasets generated and/or analyzed during the current study are not publicly available due to privacy and ethical restrictions but are available from the corresponding author on reasonable request and with the permission of the institution where the data was generated.

## Abbreviations

PPU: Perforated peptic ulcer
OS: Open surgery
LS: Laparoscopic surgery
ASA: American Society of Anesthesiologists
BMI: Body mass index.
CRP: C-reactive protein
LOS: Length of hospital stay
NLR: Neutrophil-to-lymphocyte ratio
PLR: Platelet-to-lymphocyte ratio
